# Editorial

**Published:** 1873-03

**Authors:** 


### EDITORIAL.

### The Dental Profession.

In the midst of that activity which distinguishes the dental profession, it must not be forgotten that there is yet much work to be done in extending the limits of professional knowledge, and in creating an adequate dental literature.

We have already gained a secure place among the world's vocations, but we have something yet to do to make that place an honorable one. This we are to accomplish by ridding our practice of all the characteristics of a mere calling, and by gradually imparting to it that profound acquaintance with principles, and those honorable methods which must alone distinguish the learned professions.

There are at present more talented young men of but recent connection with our body, than have ever before attached themselves to it during the same period of time. This fact is in itself a good omen. But it is true that the dentist is in many communities accorded a lower position than other professional men. In his mastery of the laws of vital phenomena, and in his acquaintance with the art of healing he may be up to the medical standard. And yet he must often find himself out-ranked by physicians who are below him in real attainment.

This state of things has led some young and ambitious dentists to entertain regrets, and to look wishfully over the wall into medical science, where the accident of profession secures a certain prestige. They have desired to wear that mantle of honor which medicine, through its great literature, long antiquity, and brilliant record is able to furnish at least to its deserving vota ies. They have found it difficult to secure their own adequate recognition in an ignorant and prejudiced community, and, in addition to this, to carry forward the standard of their profession.

That this cause of complaint is fast disappearing must be recognized by every careful observer of the times. Late brilliant achievements in the department of practice, as well as the more than respectable recent additions to our literature, have had no small share in effecting this change. It is evident that a marvelous activity of invention, ambition for achievement in practice, and enthusiasm for scientific investigation, has taken possession of our profession. Un der this stimulus, and with that sturdy vigor, which is the natural characteristic of a young profession, we shall soon take a place as a learned profession, at least not second to that of medicine.

It appears therefore that a career in dentistry should not be less provoking to the ambition of a young man of genuis than a similar career in medicine. We have yet our books to write, and our literature to create; we have yet to let in the light upon dark points in dental physiology; we have numerous problems in pathology to solve, and new departments of investigation to conduct. And if all this is insufficient there is actual practice in which scientific acquisitions and artistic skill are transmuted into brilliant results.

### The Phosphates.

As regards the ultimate cause of dental caries, there is at the present time a pretty general agreement. It is held that there are certain molecular defects incorporated into the minute structure of the teeth during their period of development, and that these structural defects make them in after life an easy prey to causes of decay that would be very trifling in their influence upon well-organized dental tissues.

As in part accounting for these defects, it is held that most civilized nations fail to take into the system a sufficient amount of tooth and bone-forming material. The enamel and dentine are thus formed under conditions which prevent a due degree of density through calcification.

The remedy for this condition in the child whose osseous tissues are still forming is found in the daily use of oat meal, barley, unbolted wheat flour, and such other vegetable andn animal aliments as contain an abundant supply of the mineral salts.

Many dental practitioners have sought to correct the deficiency of mineral salts in the blood, from which the teeth draw their nutriment, by administering doses of the phosphate of lime, which is by far the most abundant mineral ingredient of the teeth. How far this treatment has been successful is not yet known; there has been no extensive record of cases. It is, manifestly, good practice to furnish the deficient substances as far as possible through wholesome foods that contain them. At least such practice can do no harm, and is probably a more natural mode of supplying them. Oysters, beefsteak, milk, beans, peas, and barley as well as wheat, are rich in the mineral salts that are concerned in building up enamel and dentine, and should be abundantly supplied to all children.

Bolting wheat flour, for the most part, removes the mineral constituents of the grain along with the bran. It is thought that the extensive and long-continued use, of flour thus deprived of its mineral constituents, has been one cause of that general predisposition to caries which afflicts this generation.

It is very desirable that this subject should receive the attention of our thinkers and writers. Let us have articles on all the questions connected with this topic. The causes of decay, the nature of the mineral salts, the question how best to supply them, the degree of success already attained in improving dental tissues by their exhibition, and to what extent the unorganized phosphates are assimilated all need to be set forth. We need records of cases, reports of experiments, and the results of research in any and every direction.

### The Microscope.

The dental profession have found the microscope almost indispensable to a right understanding of the diseases of the teeth. This instrument is destined to play a still greater part in future courses of instruction than it has already done in the past.

It is now held that the gravest defects in the minute organization of enamel and dentine are nearly universal.

Too much study can not be given to the microscopic structure of normal tissues, and to the points wherein defectively organized teeth present differences of structure. To chemistry and the microscope we look for an explanation of many unexplored problems in dental pathology.

To the actual practitioner the microscope can not but be of very great service. It will be an eye beyond his own, to reveal conditions of which he could never have dreamed but for its aid. The microscope is as important to him who would understand and operate upon living tissues as the telescope to the astronomer. Each instrument reveals a world of its own; both reveal phenomena that would be invisible, and must have remained undiscovered, without its aid.

### New Treatment for Exposed Pulp.

Mr. Coles, of the Odontological Society, of Great Britain, proposes to treat exposed pulps with pepsin, especially if there is a suppurating surface. The pepsin digests off the diseased portion of the pulp, leaving only the healthy tissue, which is delicately capped and saved. The pepsin is to be mixed with two per cent. of hydrochloric acid, and left in the cavity three days.

More than a half century ago pepsin was used in Philadelphia as an application to cancers and indolent sores. It possesses the property of dissolving away the non living and partially disintegrated tissue, which is the source of their offensive odor. On the other hand it does not attack or even irritate those parts which retain any considerable degree of vitality.

There would seem to be one advantage to the practitioner in the pepsin treatment, in the fact that if the vitality of the pulp, or any portion of it, is very low, this portion will be dissolved along with the non-living parts. The pepsin only ceases to act at the point where it meets with vital resistance. This mode of action may derive illustration from the well-known fact that parasites in the stomach are not acted upon by the pepsin of the gastric juice so long as they live; once dead, digestion begins immediately, and they are entirely dissolved away.

If the vitality of a diseased pulp is very low, the pepsin might entirely destroy it. It appears, however, from discussions on this point in the British *Journal of Dental Science*, that entire destruction of a pulp does not result from the pepsin treatment when there is such a degree of vitality as might give a reasonable hope of saving it by capping.

There is a growing conviction on the part of our best practitioners, that such portion of the dental pulp as is healthy, or can be made so, should be preserved alive. If further experimental investigations should confirm what is already believed, pepsin may prove a valuable addition to our dental materia medica.

### Personal.

It will be interesting to the profession to know that Mr. Chas. S. Tomes, son of the eminent dentist, microscopist, and author, John Tomes, of Cavendish Square, London, is at this time on our shores. He is a young man of twenty-eight, and by his physical vigor is a living proof that the practice of dentistry in London may be so combined with lecturing before dental students as not to seriously interfere with sound health.

Mr. Tomes gave the address at the late commencement exercises of the Harvard Dental School. He has since been making hurried visits to each of the centers of dental intelligence in this country, with a view to investigate our modes of practice, and the state of dental education.

On Monday evening, March 10, Mr. Tomes had a reception by the profession of Cincinnati, at the rooms of Prof. J. Taft. After a short address by him on the state of dental education and dental legislation in England, there was a free interchange of intelligence on the various aspects of practice in the two countries. Many questions were answered on both sides, during which it appeared that the teeth in the United States are not more subject to decay than in England; that quacks are not found exclusively on this side of the Atlantic; that in London a practice can be secured through recommendations, but not easily through skill alone; that expensive contour operations are less common in England than in America; that the uniform fee for a single sitting in England, be it long or short, is one guinea, or about five dollars.

There is but one body in England who grant diplomas, namely, the College of Surgeons. They are entirely distinct from the dental hospital, which is the educating body. The student is carried through a four years' course before he is allowed to present himself for examinations.

Mr. Tomes is writing a manual of Odontology, which will be published during the current year. He intends to embark for London on the first of April. His courteous manners and evident culture are winning for him golden opinions among the members of our profession on this side of the Atlantic.

### Personal.

Dr. M. Stout, of this city, and recently of Marietta, sailed on February 1st, from San Fancisco, for Hong Kong, China, to engage in dental practice.

Very strong inducements were held out to him to go into a very large practice, which has been established by a gentleman who went out from the Ohio Dental College some seven years ago; and who has made a reputation and a success, of which any one might well be proud.

Dr. Stout leaves a large circle of friends and well wishers, both in the profession and in society. Many such fields are open, and inviting those in our profession who are thoroughly prepared to occupy them.

### Mississippi Valley Dental Association.

The twenty-ninth annual meeting of the Mississippi Valley Dental Association was held in the Ohio Dental College on the 5th, 6th, and 7th of March. It was fully as well attended as any previous meeting. Though the oldest association in the world, it is young yet in zeal and activity.

The discussions were lively and interesting; the papers presented were exceedingly good, and covered a large range of topics.

In our next number we shall give a report of papers and discussions.

### College Commencement.

The twenty-seventh annual commencement of the Ohio College of Dental Surgery was held March 4th, in College Hall, on Walnut St., in this city. The exercises were of a pleasing and interesting character. Music, both vocal and instrumental, enlivened the hour. Sixteen members of the recent class, having passed a satisfactory examination, were presented for graduation, and received the degree of Doctor of Dental Surgery, viz: John Stephan, J. Clark Scott, C. P. Dennis, B. F. Bowman, T. G. Dennis, R. J. Cunningham, S. E. Goe, F. W. Sage, H. A. Downing, R. N. Baughman, J. W. Wortman, J. Jones, Chas. H Ware, Ohio; T. S. Hacker, N. C. Thomas, Indiana; A. W. Smith, Tenn. As has ever been the custom in this institution, a neat copy of the Bible, as a rule and guide through life, accompained each diploma.

The class was addressed by Dr. Jas. Taylor, President of the Board of Trustees. The address on behalf of the faculty was delivered by Dr. Thos. Reamy, of the Miami Medical College, of this city. The valedictory on behalf of the class was delivered by B. F. Bowman, after which some delightful music closed the exercises. The next regular session of the institution will begin about the 15th of October next.

### Obituary.

Died March 5th, at his home in Troy, Ohio, Dr. B. F. Rosson, of a severe and painful illness. Dr. Rosson was well and favorably known through the profession in Ohio, He had been in practice some twenty-five years, and ever maintained a high standard of professional honor; he was always found in the right.

His loss will be severely felt by the profession, and more by the people amongst whom he dwelt, but most of all by his bereaved and sorrowing family. But the rich goodness, and memories that flow out from a well-spent life, remain for their comfort and consolation.

### The Dental Law.

Just before going to press we learn that the Legislature of this State (Ohio), has made a change in the law regulating the practice of dentistry, by which five years practice is to be regarded as a compliance with the requisitions of the law. There is a diversity of opinion as to the propriety of this change. Many were anxious to have a thorough test of the law in its original requirements. We think the law in its present shape will fail to accomplish the good it would have done as before; still in its present form it will accomplish great good for both the people and the profession.

### Local Anæsthesia.

J. H. Bill, in Braithwaites Retrospect says, "If a portion of skin is covered with a cloth, soaked with a saturated solution of carbolic acid, for half an hour, and then a streak traced across the surface, with a camel's hair pencil, dipped in the acid, liquified by one-twentieth its bulk of water, the skin may be divided along the course of the streak with a sharp scalpel, quite down to the subcutaneous and cellular tissue, without causing any pain. " The severity of the lancing in dental abscess, may be very much modified by taking advantage of this quality of carbolic acid.

### Matrimonial.

Married, on Teusday, February 11th, at St. John's Church, Baltimore Co., by Rev. L. A. Morgan, Prof. J. S. Cassidy, of Leavington, Ky., to Miss Eliza A. Guyton, of Glen Kalmia, Baltimore Co., Md.

### Longevity of the Professions.

From the observations of Dr. Grey, published some years since, it appears that gentlemen with incomes enjoy a greater longevity than any other class, and that the next most favorable positions with respect to length of life, must be accorded to medical men, the next to clergymen. What the longevity of the dental practitioner will be it remains for the future to determine, though it is probable that it will be somewhat less than that of the medical man.

### Notice.

We have received the following communication from the Committee on Literature, of the American Dental Association, and are requested to publish it:

" The Committee on Literature of the American Dental Association would request members of the profession to inform them of any new works which have appeared, or may appear, before the next annual meeting of the Association, in order that no work which should properly be considered by the Committee, need be overlooked by them."

GEO. H. CUSHING, Chairman,
550 Michigan Ave., Chicago.

E. A. BOGUE,
29 East 28th. St., New York.

C N. PIERCE.
Philadelphia.

